# Relation between myocardial blood flow and cardiac events in diabetic patients with suspected coronary artery disease and normal myocardial perfusion imaging

**DOI:** 10.1007/s12350-021-02533-w

**Published:** 2021-02-18

**Authors:** Roberta Assante, Ciro Gabriele Mainolfi, Emilia Zampella, Valeria Gaudieri, Carmela Nappi, Teresa Mannarino, Adriana D’Antonio, Parthiban Arumugam, Mario Petretta, Alberto Cuocolo, Wanda Acampa

**Affiliations:** 1 Department of Advanced Biomedical Sciences, University Federico II, Via Pansini 5, 80131 Naples, Italy; 2 Nuclear Medicine Center, Central Manchester University Teaching Hospitals, Manchester, UK; 3 Department of Translational Medical Sciences, University Federico II, Naples, Italy; 4 Institute of Biostructure and Bioimaging, National Council of Research, Naples, Italy

**Keywords:** Diabetes mellitus, Myocardial perfusion reserve, Prognosis, Diabetes mellitus, reserva de perfusión miocárdica, pronóstico

## Abstract

**Background:**

We assessed the prognostic value of structural abnormalities and coronary vasodilator function in diabetic patients referred to a PET/CT for suspected coronary artery disease (CAD).

**Methods:**

We studied 451 diabetics and 451 nondiabetics without overt CAD and normal myocardial perfusion. Myocardial blood flow (MBF) was computed from the dynamic rest and stress imaging. Myocardial flow reserve (MFR) was defined as ratio of hyperemic to baseline MBF and was considered reduced when < 2.

**Results:**

During a mean follow-up of 44 months 33 events occurred. Annualized event rate (AER) was higher in diabetic than nondiabetic patients (1.4% vs 0.3%, *P* < .001). Diabetic patients with reduced MFR had higher AER compared to those with preserved MFR (3.3% vs 0.4%, *P * < .001). At Cox analysis, age, BMI and reduced MFR were independent predictors of events in diabetic patients. Patients with diabetes and reduced MFR had lower event-free survival compared to nondiabetic patients and MFR < 2 (*P* < .001). Event-free survival was similar in patients with diabetes and normal MFR and those without diabetes and reduced MFR.

**Conclusions:**

Diabetic patients with reduced MFR had higher AER and lower event-free survival compared to those with preserved MFR and to nondiabetic patients.

**Supplementary Information:**

The online version of this article (10.1007/s12350-021-02533-w) contains supplementary material, which is available to authorized users.

## Spanish Abstract

**Antecedentes:**

Se evaluó el valor pronóstico de las alteraciones estructurales y la función vasodilatadora coronaria en pacientes diabéticos remitidos a PET/CT por sospecha de enfermedad arterial coronaria (EAC).

**Métodos:**

Se estudiaron a 451 diabéticos y 451 no diabéticos sin EAC manifiesta y perfusión miocárdica normal. Se calculó el flujo sanguíneo miocárdico (MBF) a partir de las imágenes dinámicas en reposo y estrés. La reserva de flujo miocárdico (MFR) se definió como la relación entre la MBF en hiperemia entre el basal y se consideró reducida al ser <2.

**Resultados:**

Durante un seguimiento promedio de 44 meses ocurrieron 33 eventos. La tasa anual de eventos (TAE) fue mayor en los pacientes diabéticos que en los no diabéticos (1.4% frente a 0.3%, *P* <.001). Los pacientes diabéticos con MFR reducido tuvieron una TAE más alta en comparación con aquellos con MFR preservado (3.3% vs 0.4%, *P* <. 001). En el análisis de Cox, la edad, el IMC y el MFR reducido fueron predictores independientes de eventos en pacientes diabéticos. Los pacientes con diabetes y MFR reducido tuvieron una supervivencia libre de eventos más baja en comparación con los pacientes no diabéticos y MFR <2 (*P* <. 001). La supervivencia libre de eventos fue similar en pacientes con diabetes y MFR normal y aquellos sin diabetes y MFR reducido.

**Conclusiones:**

Los pacientes diabéticos con MFR reducido tuvieron una mayor TAE y una menor supervivencia libre de eventos en comparación con aquellos con MFR conservado y con los pacientes no diabéticos.

## Chinese Abstract

**背景:**

^99m^锝-焦磷酸盐成像已成为诊断运甲状腺素蛋白心脏淀粉样变性(ATTR-CM)的一种重要的非侵入性方法。 在SPECT图像上定量分析^99m^锝-焦磷酸盐的活性可能是ATTR-CM疾病严重程度的标志。 本文评估了^99m^锝-焦磷酸盐定量分析的诊断准确性和临床意义。

**方法与结果:**

接受^99m^锝-焦磷酸盐成像的疑似ATTR-CM的患者纳入本研究。在SPECT图像中,使用心脏焦磷酸盐活性(CPA)和受累体积(VOI)计算心肌中的放射性示踪剂活性,并根据LVBP活性得出异常活性的阈值。 使用接受者操作特征曲线下的面积(AUC)评估诊断准确性。

本实验共入组124例患者,平均年龄为73±11.4,其中43例(34.7%)患者被诊断为ATTR-CM。 CPA的诊断准确度最高(AUC 0.996,95%CI 0.987 - 1.00),与Perugini评分相比明显更高(AUC 0.952,p = 0.016)。 在ATTR-CM的患者中,CPA与左心室射血分数降低(调整后的优势比1.28,p = 0.035)和心衰住院(调整后的危险比1.29,p = 0.006)相关。

**结论:**

使用CPA或VOI定量评估心肌放射性示踪剂的活性对ATTR-CM具有很高的诊断准确性。 两种方法都是潜在的非侵入性指标,可跟踪疾病的进展或治疗效果。

## French Abstract

**Contexte:**

Nous avons évalué la valeur pronostique des anomalies structurelles et de la vasodilatation coronaire chez les patients diabétiques qui nous ont été envoyé pour un examen TEP/TDM pour suspicion de maladie artérielle coronaire (MAC).

**Méthodes:**

Nous avons étudié 451 diabétiques et 451 non-diabétiques sans MAC manifeste et avec une perfusion myocardique normale. Le débit sanguin myocardique (DSM) a été calculé sur bases des images TEP/TDM de repos et de stress. La réserve de perfusion myocardique (RPM) a été définie comme le rapport entre l’hyperémie myocardique et la perfusion myocardique de base et a été considérée comme réduite avec un rapport inferieur à 2.

**Résultats:**

Au cours d’un suivi moyen de 44 mois, 33 événements cardiaques ont été enregistrés. Le nombre d’événements cardiaques annualisés (ECA) s’est avéré plus élevé chez les patients diabétiques que chez les patients non diabétiques (1,4% vs 0,3%, P <0,001). Les patients diabétiques dont la RPM était réduite ont montré un nombre d’ECA plus élevé que ceux dont la RPM était préservée (3,3% vs 0,4%, P <0,001). L’analyse statistique basée sur la méthode Cox a montré que, l’âge, l’IMC et la réduction de la RPM sont des paramètres indépendants chez les patients diabétiques. Les patients atteints de diabète avec une réduction de leur RPM ont eu une survie avec un nombre plus élevés d’événements cardiaques par rapport aux patients non diabétiques avec une RPM inferieure à 2 (P <.001). La survie sans événements cardiaques s’est révélée similaire chez les patients atteints de diabète avec une RPM normale et ceux sans diabète avec une RPM réduite.

**Conclusions:**

Dans cette étude, les patients diabétiques avec un MFR réduit ont eu une survie entachée d’un nombre plus important d’événements cardiaques par rapport aux diabétiques avec une réserve de perfusion myocardique préservée et aux patients non diabétiques.

## Introduction

Diabetes mellitus is associated with higher cardiovascular mortality compared to general population. This aspect could be also related to correlation of diabetic status with cardiovascular risk factors that may contribute to increased risk for coronary artery disease (CAD).^1^ However, beyond traditional risk factors other mechanisms are involved in the increased cardiovascular risk among patients with diabetes, such as the effect of the disease on endothelial function.^2^ In diabetic patients there is an evidence of structural and functional alterations of coronary arteries and diffuse atherosclerosis process, which may improve the prediction of cardiovascular events. In particular, diffuse atherosclerosis is associated with higher prevalence of impaired myocardial flow reserve (MFR), reflecting both the presence of epicardial coronary artery stenosis and microvascular dysfunction.^3^ Reduced MFR is associated with higher rate of cardiac mortality in diabetic patients with and without known CAD, demonstrating that probably microvascular dysfunction may be an early manifestation of CAD.^4^ In addition, coronary artery calcium (CAC), an index of atherosclerotic burden, is higher in diabetic subjects.^5^,^6^ Despite the presence of inverse relationship between MFR and CAC score in a general population,^7^ a recent study demonstrated that diabetic patients had lower MFR regardless all CAC score categories as compared to nondiabetics, showing that the two process were different entities.^8^ The prognostic impact of microvascular dysfunction and atherosclerosis has been evaluated in subjects with suspected CAD referred to CAC scoring and MFR evaluation, as a part of the same examination by positron emission tomography (PET)/computed tomography (CT).^9^,^10^ It has been demonstrated that at any level of severity of coronary calcification, impaired MFR identified patients at higher short-term risk of adverse cardiac events.^9^ Only few studies investigated the prognostic rule of coronary vascular function and atherosclerosis burden in diabetic patients without overt CAD. The aim of our study was to evaluate the prognostic value of measures of structural abnormalities and coronary vasodilator function in diabetic patients referred to a PET/CT study for suspected CAD and showing normal myocardial perfusion.

## Methods

### Patient Population

We studied 3974 consecutive patients referred to PET/CT as a part of their diagnostic work-up. Patients (*n* = 1920) have been excluded for: (1) documented history of CAD defined as luminal stenosis > 50% at coronary angiography, previous percutaneous coronary intervention, coronary artery bypass graft surgery or myocardial infarction; (2) uncontrolled atrial fibrillation, pacemaker or prosthetic valve. Sixty-three patients were excluded for the presence of abnormal myocardial perfusion imaging (MPI). Among the remaining 1991 patients enrolled, 502 (25%) had a history of type-2 diabetes and 1489 (75%) did not. Diabetes was defined when the patients had any one of the criteria as follows: fasting blood glucose ≥ 126 mg/dL, random blood glucose ≥ 200 mg/dL, blood glucose ≥ 200 mg/dL 2 h after a 75 g oral glucose tolerance test within the past 3 months, currently taking drugs to treat hyperglycemia, or prior medical diagnosis of diabetes. Diabetic patients were defined as under control in the presence of the HbA1c values between 6.5% and 7.5%.^11^ As part of the baseline examination, clinical teams collected information on traditional cardiovascular risk factors (including age, sex, body mass index, dyslipidemia, smoking, hypertension, family history of CAD). Hypertension was defined as a blood pressure ≥ 140/90 mmHg or the use of anti-hypertensive medication.^12^ Hypercholesterolemia was defined as total cholesterol level ≥ 6.2 mmol/L or treatment with cholesterol lowering medication. A positive family history of CAD was defined by the presence of disease in first-degree relatives younger than 55 years in men or 65 years in women. The review committee of the institution approved the study and all patients gave informed consent (Ethics Committee, University Federico II, protocol number 110/17).

### PET Imaging

As a routine preparation for ^82^Rb cardiac PET/CT, patients were asked to discontinue taking nitrates for 6 hours, calcium channel blockers and caffeine-containing beverages for 24 hours, and b-blockers for 48 hours before their appointment. Scans were acquired using a Biograph mCT 64-slice scanner (Siemens Healthcare). Rest and stress cardiac PET/CT images were acquired as follows: scout CT to check the patient position and low-dose CT (0.4 mSv; 120 kVp; effective tube current, 26 mA [11-mAs quality reference]; 3.3 s) were performed for attenuation correction, during normal breathing before and after PET acquisitions. For both rest and stress images 1110 MBq of ^82^Rb were injected intravenously and a 6-minute list-mode PET study was acquired. Pharmacologic stress was then administered using adenosine (140 μg × kg^−1^ × min^−1^ for 4.5 min). Both rest and stress dynamic images were reconstructed into 26-time frames (12 × 5 seconds, 6 × 10 seconds, 4 × 20 seconds, and 4 × 40 seconds; total, 6 minutes) using the vendor standard ordered-subsets expectation maximization 3D reconstruction (2 iterations, 24 subsets) with 6.5-mm gaussian post-processing filter. Regional myocardial perfusion was visually assessed, using standardized segmentation of 17 myocardial regions.^13^ Each myocardial segment was scored from normal (score = 0) to absent perfusion (score = 4). The summed stress score was obtained by adding the scores of the 17 segments of the stress images. The same procedure was applied to the resting images to calculate the summed rest score and summed difference score was the difference between the stress and rest scores. Myocardial perfusion was considered normal when summed stress score was < 3. Absolute myocardial blood flow (MBF) (in mL × min^−1^ × g^−1^) was computed from the dynamic rest and stress imaging series with commercially available software (Siemens Syngo Dynamic PET).^14^ MFR was defined as the ratio of hyperemic to baseline MBF and was considered reduced when < 2.^15^ The MFR values were calculated using baseline MBF corrected for rate-pressure product (RPP). Adjusted values were computed by using an automatic software as MBF_adj_ = MBF_rest_/RPP_rest_ × RPP_ref_, where RPP_ref_ is the reference value of 8500 reported for a typical CAD population.^16^

### CT Imaging

All patients underwent a CT scan for CAC scoring. Those with heart rate > 75 bpm received prior intravenous beta-blockers (5-10 mg atenolol). A standard scanning protocol was applied, with 18 mm section collimation (30 × 0.6 mm), 0.24 ms gantry rotation time, 120 kVp tube voltage and 60 Q ref mAs tube current. CAC scoring was obtained during a single breath hold and coronary calcification was defined as a plaque with an area of 1.03 mm2 and a density ≥ 130 HU. The CAC score was calculated according to the method described by Agatston.^17^ Experienced nuclear medicine physicians analyzed the CT, blinded to the PET results (Siemens, Syngo Multimodality Workplace). The CAC score was categorized into two groups (< 400 and ≥ 400).

### Follow-up

Patient follow-up was prospectively obtained by use of a questionnaire that was assessed by a phone call to all patients and general practitioners or cardiologists and by review of hospital or physicians’ records by individuals blinded to the patient’s test results. The outcome was a composite end-point of cardiac death, nonfatal myocardial infarction, or unstable angina requiring coronary revascularization, whichever occurred first. The cause of death was confirmed by review of death certificate, hospital chart, or physician’s records. Death was considered to be of cardiac origin if the primary cause was defined as acute myocardial infarction, congestive heart failure, valvular heart disease, sudden cardiac death, or cardiac interventional/ surgical procedure related. Myocardial infarction was defined when > 2 of the following 3 criteria were met: chest pain or equivalent symptom complex, positive cardiac biomarkers, or typical electrocardiographic changes.^18^ The date of the last examination or consultation was used to determine the length of follow-up.

### Statistical Analysis

Continuous data are expressed as mean ± standard deviation and categorical data as percentage. A student two-sample t-test and chi-square test were used to compare the differences in continuous and categorical variables, respectively. A *P* value < .05 (two-sided) was considered statistically significant. To create a matched cohort of diabetic

and nondiabetic patients, a propensity score (logit model) was calculated for each individual based on the baseline clinical variables (age, sex, body mass index, hypertension, hypercholesterolemia, smoking history and family history of CAD). A one-to one matched analysis without replacement was performed on the basis of the estimated propensity score of each patient.^19^ To perform the matching and to select the final dataset for analysis, the nearest available Mahalanobis metric matching method with caliper size specification (0.25 × SD of propensity score) was used. After propensity score matching, baseline characteristics were compared. In addition, the success of propensity score matching was assessed using standardized differences.^20^ Propensity score analyses were conducted using the Stata module PSMATCH2.^21^

Annualized event rate (AER), expressed as % person-years, was calculated as the cumulative number of events divided by person-time. This latter is an estimate of the actual time-at risk that all persons contribute to the study, i.e., the sum of each individual follow-up period. Hazard ratios with 95% confidence intervals (CI) were calculated by univariable and multivariable Cox regression analysis. Variables showing a *P* value < .05 at univariable analysis were considered for multivariable analysis. The incremental prognostic value of MFR over clinical variables and calcium scoring was assessed based on the log-likelihood ratio chi-square statistic, adjusted for differences in degree of freedom. Event-free survival curves were obtained by the Kaplan-Meier method and compared with the log-rank test. A parametric survival model was used to identify how the variables influenced time to event and to estimate cumulative hazard during the follow-up. Among the parametric models currently used in survival analysis, Weibull model resulted as having the best fit based on the Akaike information criterion and was chosen to perform the analysis. The appropriateness of the Weibull model was also graphically assessed, showing a straight line plotting the log (-log) of the estimated baseline survival function against log time. Statistical analysis was performed with Stata 14 software (StataCorp, College Station, Texas USA).

## Results

Of the 1991 patients enrolled, follow-up data were not available in 149 patients (7%), leaving 1842 subjects for the analysis. Among these latter patients, 452 (24%) had a history of type-2 diabetes and 1390 (76%) did not. All diabetic patients were in controlled condition, showing HbA1c values between 6.5% and 7.5%. Patients clinical characteristics and imaging findings in relation to diabetic status are described in Table 1. As shown, before propensity score diabetic patients showed higher body mass index (BMI) and higher prevalence of male gender, hypertension, dyslipidemia, and family history of CAD compared to nondiabetic patients. Moreover, diabetic patients had lower value of hyperemic MBF and MFR (both *P* < .001) compared to nondiabetic patients, while baseline MBF was similar. Diabetic patients also showed a higher prevalence of CAC ≥ 400 (14% vs 9%, *P < *.001) compared to nondiabetic patients. After propensity matching all clinical characteristics were comparable between 451 diabetic and 451 nondiabetic patients. Yet, diabetic patients still had lower hyperemic MBF and MFR than nondiabetic patients, while the CAC scores and baseline MBF were not significantly different. The number of patients with reduced MFR did not increased in the nondiabetic cohort after matching, despite the raise in the percentage of risk factors in this group. In the matched cohort, diabetes (*P < *.001), age (*P < *.001) and hypertension (< .05) resulted independent predictors of reduced MFR.

**Table 1:** Clinical characteristics and imaging findings by diabetic status before and after propensity score matching

	Before matching			After matching		
	Diabetes (*n* = 452)	No diabetes (*n* = 1390)	*P* value	Diabetes (*n* = 451)	No diabetes (*n* = 451)	*P* value
Age (years)	59 ± 12	58 ± 13	.08	59 ± 12	60 ± 12	.26
Male gender, *n* (%)	218 (48)	582 (42)	< .05	218 (48)	230 (51)	.42
Body mass index (kg/m^2^)	32 ± 7	30 ± 7	< .001	32 ± 7	32 ± 7	.62
Hypertension, *n* (%)	370 (82)	893 (64)	< .001	370 (82)	376 (83)	.60
Dyslipidemia, *n* (%)	353 (78)	765 (55)	< .001	353 (78)	354 (78)	.94
Smoking history, *n* (%)	82 (18)	277 (20)	.41	82 (18)	94 (21)	.31
Family history of CAD, *n* (%)	190 (42)	683 (49)	< .05	190 (42)	191 (42)	.95
Baseline MBF (ml/g/min)	1.06 ± .34	1.09 ± 0.37	.18	1.06 ± 0.34	1.07 ± 0.38	.64
Hyperemic MBF (ml/g/min)	2.52 ± 0.80	2.80 ± 0.79	< .001	2.52 ± 0.80	2.76 ± 0.80	< .001
MFR	2.46 ± 0.70	2.68 ± 0.71	< .001	2.46 ± 0.70	2.69 ± 0.71	< .001
MFR < 2, *n* (%)	124 (27)	236 (17)	< .001	124 (27)	70 (17)	< .001
CAC categories						
< 400, *n* (%)	388 (86)	1269 (91)	< .001	387 (86)	397 (88)	.32
≥ 400, *n* (%)	64 (14)	121 (9)	< .001	64 (14)	54 (12)	.32

Values are expressed as mean value ± standard deviation or as number (percentage) of subjects

*CAD*, coronary artery disease; *MBF*, myocardial blood flow; *MFR*, myocardial flow reserve; *CAC*, coronary artery calcium

### Patient Outcome

**Figure 1:**
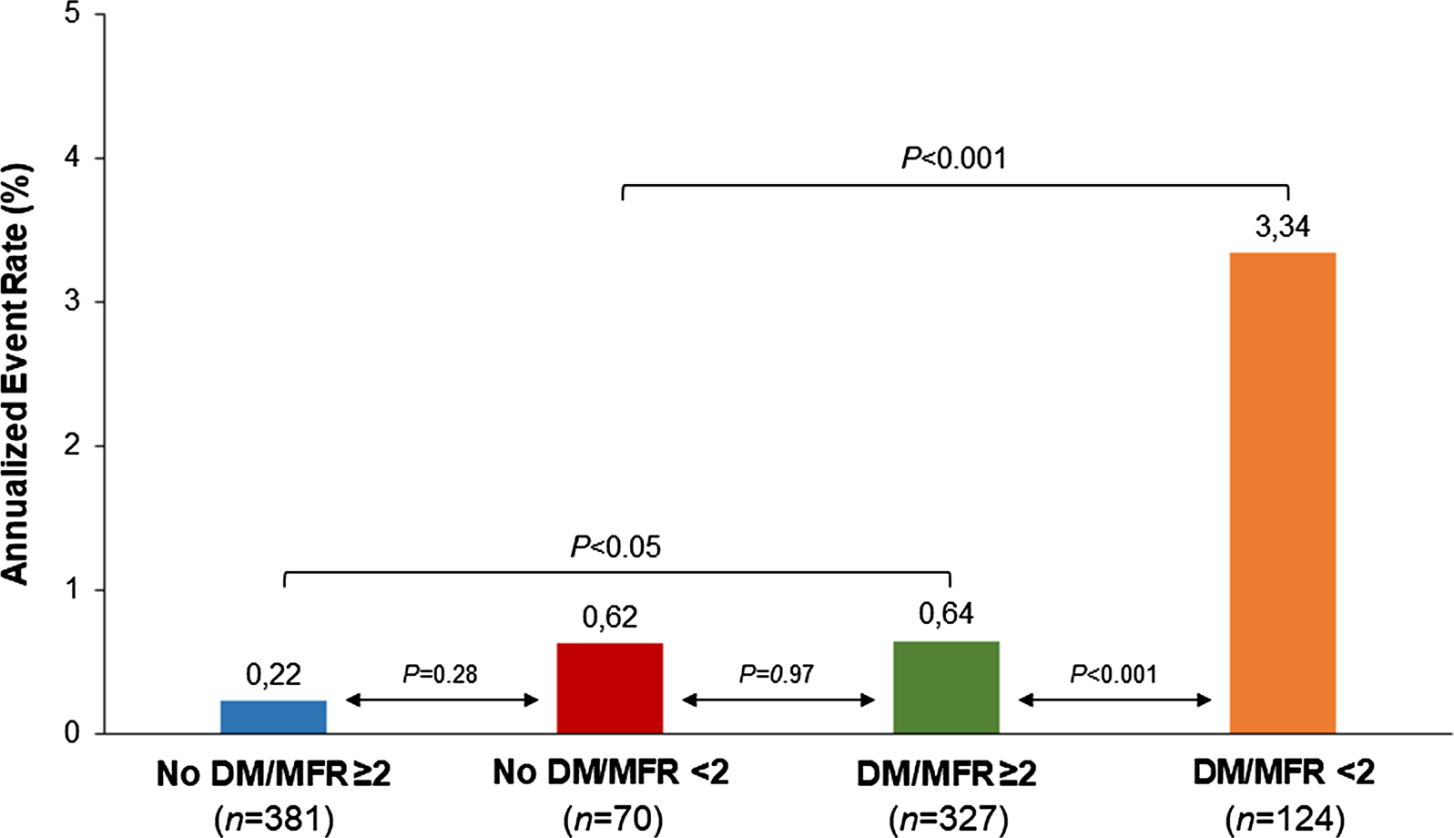
Annualized event rate according to diabetes status and MFR

In the propensity score-matched cohort during follow-up (median 44 months, interquartile range 4-106), 33 events occurred (3.7% cumulative event rate, with an annual event rate of 0.8% person-years). The events were cardiac death in 10 patients, nonfatal myocardial infarction in 12, and unstable angina requiring revascularization in 11. AER was higher for diabetic than nondiabetic patients (1.4% vs 0.3%, *P* < .001). AER according to diabetes and MFR are depicted in Figure 1. As shown, there were no significant differences in AER in no diabetic patients with preserved or reduced MFR (*P* = .28). On the contrary, diabetic patients with reduced MFR showed a higher event rate as compared to those with preserved MFR (*P <* .001). In patients with reduced MFR, presence of diabetes was also associated with higher event rate (*P <* .001). Of note, nondiabetic patients with reduced MFR and diabetic patients with preserved MFR showed similar values of annualized event rate (*P* = .97).

### Predictors of Events

Results of Cox regression analysis in the mixed population of diabetic and nondiabetic patients, both before and after matching, are reported in Table 2 and Table 3, respectively. Before matching, age, male gender, diabetes, and reduced MFR were independent predictors of events. At incremental analysis the addition of MFR data to clinical variables and CAC score increased the global chi-square of the model from 58 to 76 (*P* < .001). After matching, age, male gender, diabetes, and reduced MFR resulted independent predictors of event. The addition of MFR data to clinical variables and CAC score increased the global chi-square of the model from 32 to 54 (*P* < .001).

**Table 2:** Univariable and multivariable predictors of adverse cardiac events in the overall population before matching

	Univariable analysis		Multivariable analysis	
	Hazard ratio (CI)	*P* value	Hazard ratio (CI)	*P* value
Age	1.041 (1.016-1.066)	< .005	1.033 (1.007-1.060)	< .05
Male gender	2.262 (1.249-4.097)	< .05	2.212 (1.203-4.066)	< .05
Body mass index	0.976 (0.933-1.022)	.31		
Diabetes mellitus	4.910 (2.726-8.846)	< .001	3.988 (2.168-7.336)	< .001
Hypertension	0.448 (0.216-0.932)	< .05	0.898 (0.418-1.932)	.78
Dyslipidemia	0.587 (0.309-1.117)	.10		
Smoking history	0.962 (0.464-1.993)	.91		
Family history of CAD	0.888 (0.498-1.585)	.68		
MFR < 2	4.285 (2.398-7.657)	< .0001	3.097 (1.705-5.624)	< .001
CAC score ≥ 400	2.777 (1.409-5.472)	< .05	1.242 (0.604-2.553)	.57

*CI,* confidence interval; *CAD*, coronary artery disease; *MFR*, myocardial flow reserve*; CAC*, coronary artery calcium

**Table 3:** Univariable and multivariable predictors of adverse cardiac events in the overall population after matching

	Univariable analysis		Multivariable analysis	
	Hazard ratio (CI)	*P* value	Hazard ratio (CI)	*P* value
Age	1.035 (1.005-1.065)	< .05	1.032 (1.002-1.063)	< .05
Male gender	2.657 (1.258-5.613)	< .05	2.913 (1.357-6.252)	< .05
Body mass index	0.946 (0.894-1.002)	0.06		
Diabetes mellitus	4.709 (1.942-11.420)	< .005	4.129 (1.680-10.146)	< .005
Hypertension	1.151 (0.498-2.661)	0.74		
Dyslipidemia	1.238 (0.557-2.750)	0.60		
Smoking history	0.858 (0.372-1.978)	0.71		
Family history of CAD	1.277 (0.631-2.587)	0.49		
MFR < 2	5.488 (2.722-11.064)	< .001	4.157 (2.041-8.470)	< .001
CAC score ≥ 400	1.643 (0.712-3.791)	0.24		

*CI,* confidence interval; *CAD*, coronary artery disease; *MFR*, myocardial flow reserve*; CAC*, coronary artery calcium

**Figure 2:**
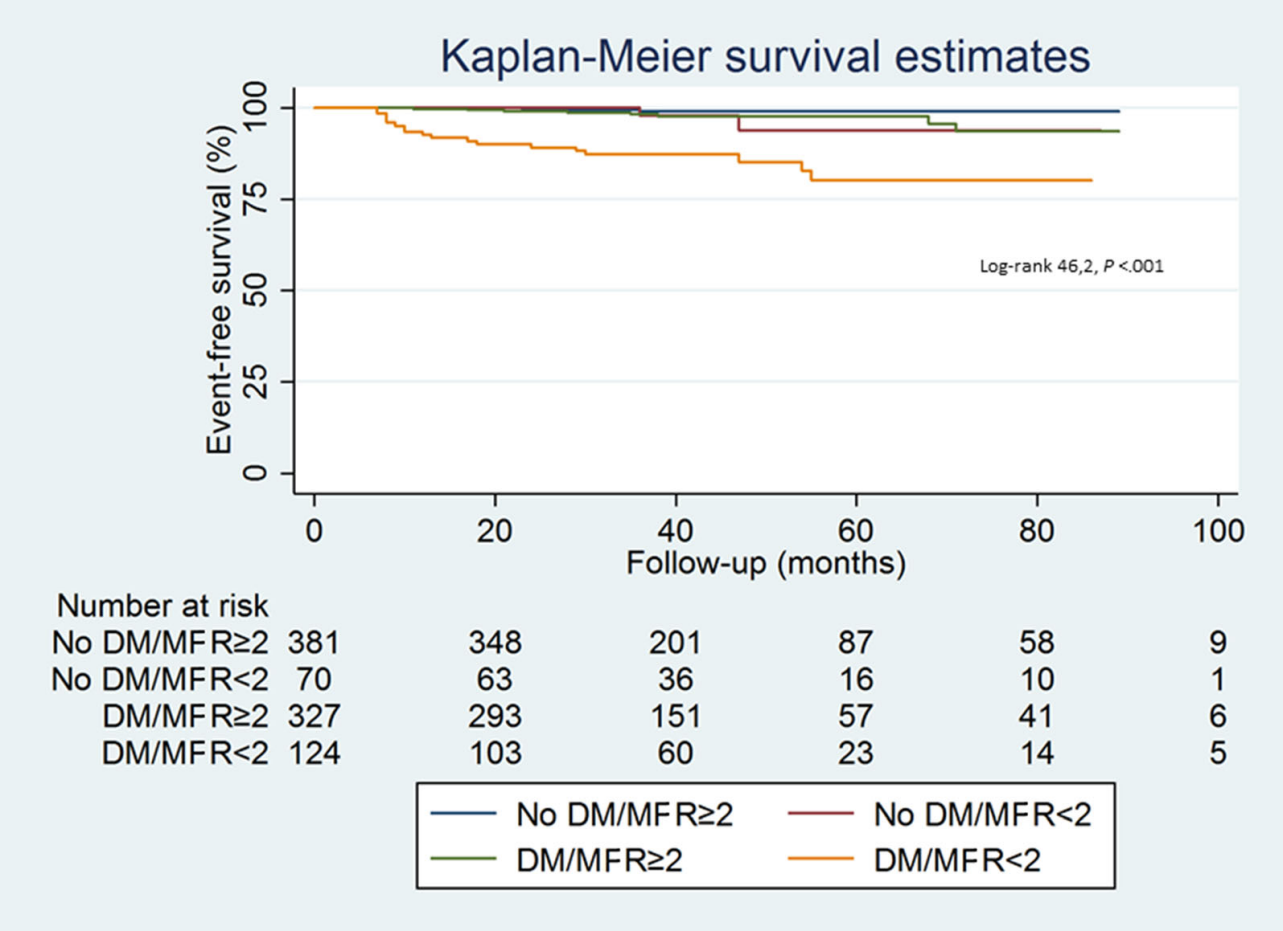
Kaplan-Meier event-free survival curves according to diabetes status and MFR

Results of Cox regression analysis performed separately in diabetic and nondiabetic patients after propensity score matching are reported in Table 4. Age, BMI, male gender, and reduced MFR were univariable predictors of events in diabetic patients, while no predictors of events resulted in nondiabetic patients. At multivariable analysis, age (*P* < .01), BMI (*P* < .05), and reduced MFR (*P* < .001) were independent predictors of events only in diabetic patients. The event-free survival curves according to diabetes and MFR status are reported in Figure 2. Patients with diabetes and reduced MFR showed the worst outcome; in particular, they had lower event-free survival as compared to nondiabetic patients with MFR < 2 (*P* < .001). Interestingly, event-free survival was similar in patients with diabetes and normal MFR and those without diabetes and reduced MFR (*P *= .89). The best outcome was observed in nondiabetic patients with MFR ≥ 2.

**Table 4:** Univariable predictors of adverse cardiac events in patients with and without diabetes

	Diabetes		No Diabetes	
	Hazard ratio (CI)	*P* value	Hazard ratio (CI)	*P* value
Age	1.054 (1.018-1.090)	< .005	0.983 (0.924-1.046)	.59
Male gender	2.296 (1.029-5.125)	< .05	7.094 (0.798-63.045)	.08
Body mass index	0.934 (0.875-0.996)	< .05	0.971 (0.854-1.103)	.65
Hypertension	0.971 (0.367-2.568)	.95	1.926 (0.340-10.897)	.46
Dyslipidemia	1.382 (0.583-3.276)	.46	0.661 (0.075-5.798)	.71
Smoking history	1.071 (0.369-3.112)	.90	0.295 (0.059-1.472)	.14
Family history of CAD	1.334 (0.603-2.951)	.48	1.387 (0.254-7.578)	.71
MFR < 2	5.126 (2.296-11.448)	< .0001	3.171 (0.057-1.743)	.19
CAC score ≥ 400	2.040 (0.862-4.826)	.11	2.720 (0.900-8.216)	.54

*CI,* confidence interval; *CAD*, coronary artery disease; *MFR*, myocardial flow reserve*; CAC*, coronary artery calcium

### Change in Risk With Time

**Figure 3:**
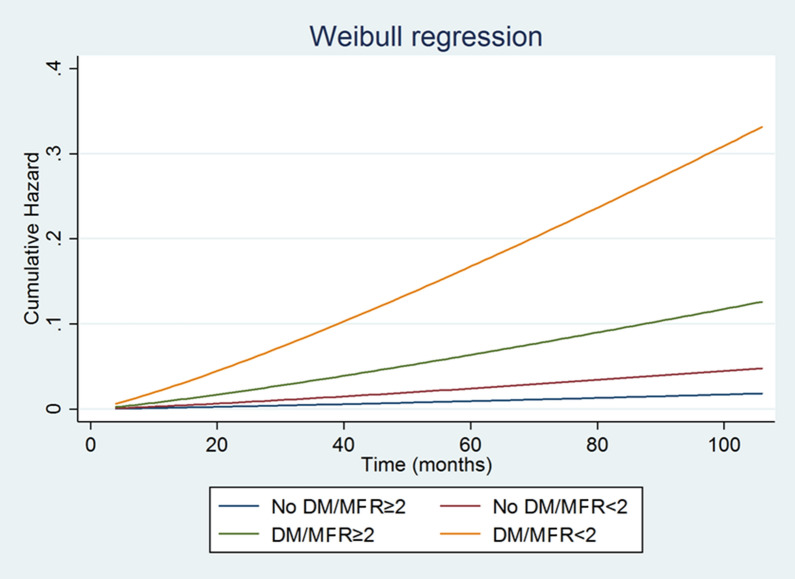
Estimated probability of cardiac events according to diabetes status and MFR

The predicted cumulative hazard according to diabetes and MFR status is depicted in Figure 3. Parametric survival analysis including in the model diabetes and MFR revealed that the highest risk of cardiac events and the major risk acceleration was observed in diabetic patients with reduced MFR. Conversely, nondiabetic patients with preserved MFR had the lowest probability of events. The probability of events was comparable in nondiabetic patients with reduced MFR and diabetic patients with preserved MFR.

Estimated time to achieve a cumulative cardiac risk level (3%) in diabetic and nondiabetic patients according to MFR showed that nondiabetic patients with normal MFR remained at low risk for the length of follow-up, while in diabetic patients with reduced MFR the time to achieve a risk level of events 3% was 12 months. The time to achieve a risk 3% was similar in nondiabetic patients with reduced MFR and diabetic patients with normal MFR (after 38 months and 45 months, respectively).

## Discussion

Diabetes represents an increasing problem and cardiovascular disease is the most common cause of death in diabetic population.^1^ It has demonstrated that the higher prevalence of cardiovascular risk factors would probably taking into account for the high cardiovascular mortality.^22^ However, in diabetics other important factors could affect the probability of cardiovascular disease. Both macro- and microvasculature involvement in diabetic cardiovascular disease have been demonstrated.^23^ In detail, previous studies found that the presence of diffuse atherosclerotic as high CAC content and endothelium dysfunction by reduced MFR are most prevalent in diabetic patients.^3^ Recently, it has been shown that in patients with diabetes coronary atherosclerosis burden and vascular function are two different entities with different etiology.^8^ In particular, diabetic patients with suspected CAD evaluated by PET/CT showed a higher CAC content than nondiabetic patients, but the difference disappeared when clinical characteristic were taken into account by propensity score. Moreover, diabetic patients had lower MFR regardless of CAC score category than nondiabetic patients.^8^ These findings were confirmed in the present patient population in which we aimed to evaluate the prognostic value of coronary vascular dysfunction comparing diabetic and nondiabetic patients with suspected CAD and normal MPI. In agreement with previous studies, we found a higher prevalence of cardiac events in diabetic compared to nondiabetic patients. Moreover, diabetic patients with reduced MFR showed higher AER as compared those with preserved MFR. Recently, in a population of 436 patients with low-intermediate risk of CAD it has been demonstrated that both the extent of coronary atherosclerotic burden and the presence of coronary vascular dysfunction are associated with increased risk of adverse cardiac events during a long-term follow-up, even after adjustment for cardiovascular risk factors.^9^ These findings that direct measures of coronary vasodilator function may be more powerful measures of CAD risk than simply the total burden of calcified atherosclerosis helping to stratify patients with low-intermediate risk of CAD.

Naya et al^24^ in 901 consecutive patients with suspected CAD and normal MPI showed that MFR but not CAC provides significant incremental risk stratification over clinical risk score for prediction of major adverse cardiac events. However, no separate analysis in diabetic patients has been performed to assess the prognostic role of imaging variables in this cohort. Prognostic role of the presence of coronary vascular dysfunction in patients with suspected or known CAD have been already investigated.^25^-^27^ A recent metanalysis confirmed that in patients with suspected or known CAD, an impaired MFR is associated with adverse cardiovascular events.^28^ Murthy et al^4^ in a population of 2783 patients with suspected or known CAD and including both normal and abnormal MPI findings, found that the presence of coronary vascular dysfunction was associated with higher probability of events in both diabetic and nondiabetics patients, improving risk stratification, in a follow-up period of 1.4 years. Importantly, the authors demonstrated that diabetic patients without known CAD with impaired coronary vascular function experienced a rate of cardiac death comparable to, and possibly higher than that for nondiabetic patients with known CAD. As compared to previous published data 4,24 in our study we evaluated only diabetic and nondiabetic patients with suspected CAD and normal MPI in a longer follow-up period of about 4 years. From our data it emerged that impaired MFR was able to identify diabetic patients at higher risk of events. Moreover, nondiabetic patients with reduced MFR and diabetic patients with preserved MFR showed similar values of annualized event rate, confirming that presence of diabetes also with preserved MFR represents a strong prognostic factor. It has already been demonstrated that diabetes is an important predictor of hard event in patients with suspected and known CAD.^29^ It has also been demonstrated that diabetic patients with normal MPI are at higher risk of cardiac events with a variable warranty period.^30^ Thus, from this study it emerged the possibility of further stratify by MFR in the presence of diabetes. Considering that impairment of MBF in diabetic patients may be an important reason for the higher event rates, aggressive statin therapy in such patients would be important also in term of primary prevention. Moreover, from our data it emerged that diabetic patients with preserved MFR have the same event rate as nondiabetics with reduced MFR. These results support the concept of CAD equivalence for diabetic patients and confirm that after a normal stress MPI, diabetic patients are at higher risk for cardiac events than nondiabetics also after balancing clinical characteristics and stress type by propensity score analysis.^30^,^31^ Recent data also confirmed that diabetic patients with normal MPI carried similar risk to nondiabetic patients with abnormal study.^32^ Presence of cardiac risk factors such as dyslipidemia associated with endothelial dysfunction contributes to promote and accelerates atherosclerosis. In the present study, to overcome bias deriving from the presence of others cardiovascular risk factors, we used a propensity score-matched analysis of a cohort of diabetic and nondiabetic patients with normal MPI findings. As already demonstrated in a previous paper,^8^ we found that after propensity matching diabetic patients still have lower values of MFR, while CAC score values were not different compared to nondiabetic patients. Interestingly, the number of patients with MFR < 2 did not increase in the nondiabetic cohort after matching, despite the increase in the percentage of patients with hypertension and dyslipidemia. Thus, controlled hypertension seems to have no impact on the number of patients with reduced MFR in the absence of diabetes. This result supports the notion that coronary risk factors have a synergistic and multiplicative effect rather than a simply additive effect on global cardiovascular risk.

In the present study, after matching patients according to clinical variables, we found that diabetic patients with reduced MFR showed the worst outcome and the major risk acceleration at parametric analysis, while nondiabetic patients with preserved MFR had the lowest probability of events. This acceleration confirms the need to identify subset of patients who can derive the most benefit from the intensive prevention measures with aggressive risk factor modification and a retesting strategy. Noninvasive measure of coronary vascular dysfunction could represent a powerful predictor of adverse cardiac events and may helpful to better stratify diabetic patients. The implications of our observations deserve further investigation and specifically, whether impaired MFR can identify diabetics who will benefit from targeted medical interventions. Unfortunately, in the present investigation angiographic data were not available. Thus, despite our population was characterized by subjects at low-intermediate risk of CAD and normal MPI we cannot exclude that presence of significant CAD associated with microvascular disease may have influenced the cardiac event rates.

## New knowledge gained

The results of this study add new information about the prognostic value of coronary vascular dysfunction by ^82^Rb cardiac PET/CT in diabetic patients with suspected CAD and normal MPI. In particular, the study highlighted that noninvasive measure of coronary vascular dysfunction by MFR may help to better stratify diabetic patients.

## Conclusion

Diabetic patients with reduced MFR showed a higher event rate and lower event-free survival compared to those with preserved MFR and to nondiabetic patients with preserved or reduced MFR. Interestingly, event rate and event-free survival was similar in patients with diabetes and normal MFR and subjects without diabetes and reduced MFR.

## Supplementary Information

Below is the link to the electronic supplementary material.

Supplementary material 1 (PPTX 342 kb)

Supplementary material 2 (M4a 4190 kb)

## Abbreviations

CAC: Coronary artery calcium
CAD: Coronary artery disease
MFR: Myocardial flow reserve
MBF: Myocardial blood flow
PET: Positron emission tomography
CT: Computed tomography
MPI: Myocardial perfusion imaging
AER: Annualized event rate
BMI: Body mass index
