# Supplementary material for: Relation between myocardial blood flow and cardiac events in diabetic patients with suspected coronary artery disease and normal myocardial perfusion imaging
Source: J Nucl Cardiol. 2021 Feb 18;28(4):1222–33. doi: 10.1007/s12350-021-02533-w (PMC8421293; doi:10.1007/s12350-021-02533-w)
Supplement: Supplementary file 1 — Supplementary material 1 (PPTX 342 kb) [file 12350_2021_2533_MOESM1_ESM.pptx]

## Slide 1
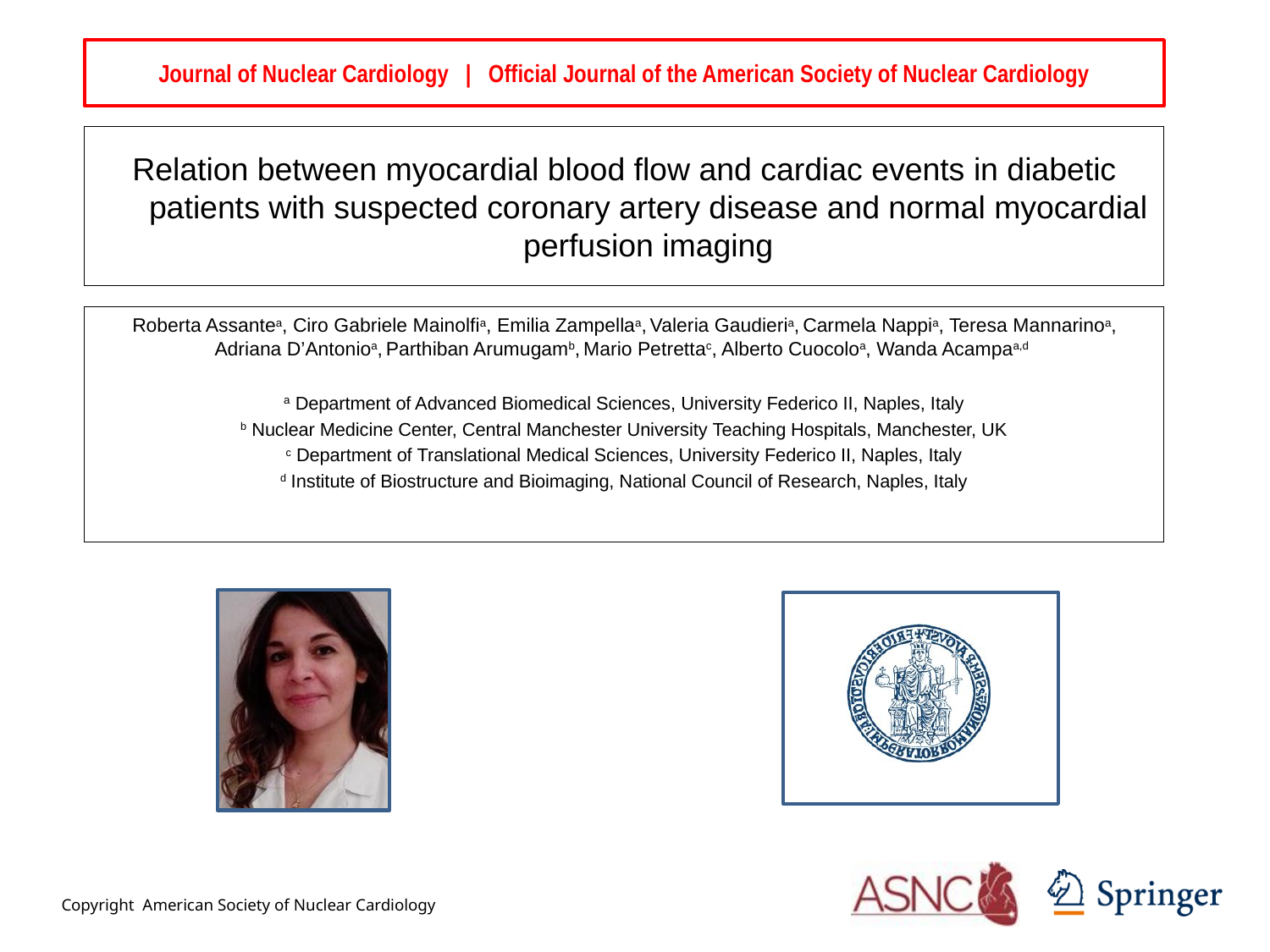

Journal of Nuclear Cardiology | Official Journal of the American Society of Nuclear Cardiology
# Relation between myocardial blood flow and cardiac events in diabetic patients with suspected coronary artery disease and normal myocardial perfusion imaging
Roberta Assantea, Ciro Gabriele Mainolfia, Emilia Zampellaa, Valeria Gaudieria, Carmela Nappia, Teresa Mannarinoa, Adriana D’Antonioa, Parthiban Arumugamb, Mario Petrettac, Alberto Cuocoloa, Wanda Acampaa,d
a Department of Advanced Biomedical Sciences, University Federico II, Naples, Italy
b Nuclear Medicine Center, Central Manchester University Teaching Hospitals, Manchester, UK
c Department of Translational Medical Sciences, University Federico II, Naples, Italy
d Institute of Biostructure and Bioimaging, National Council of Research, Naples, Italy
Copyright American Society of Nuclear Cardiology

## Slide 2
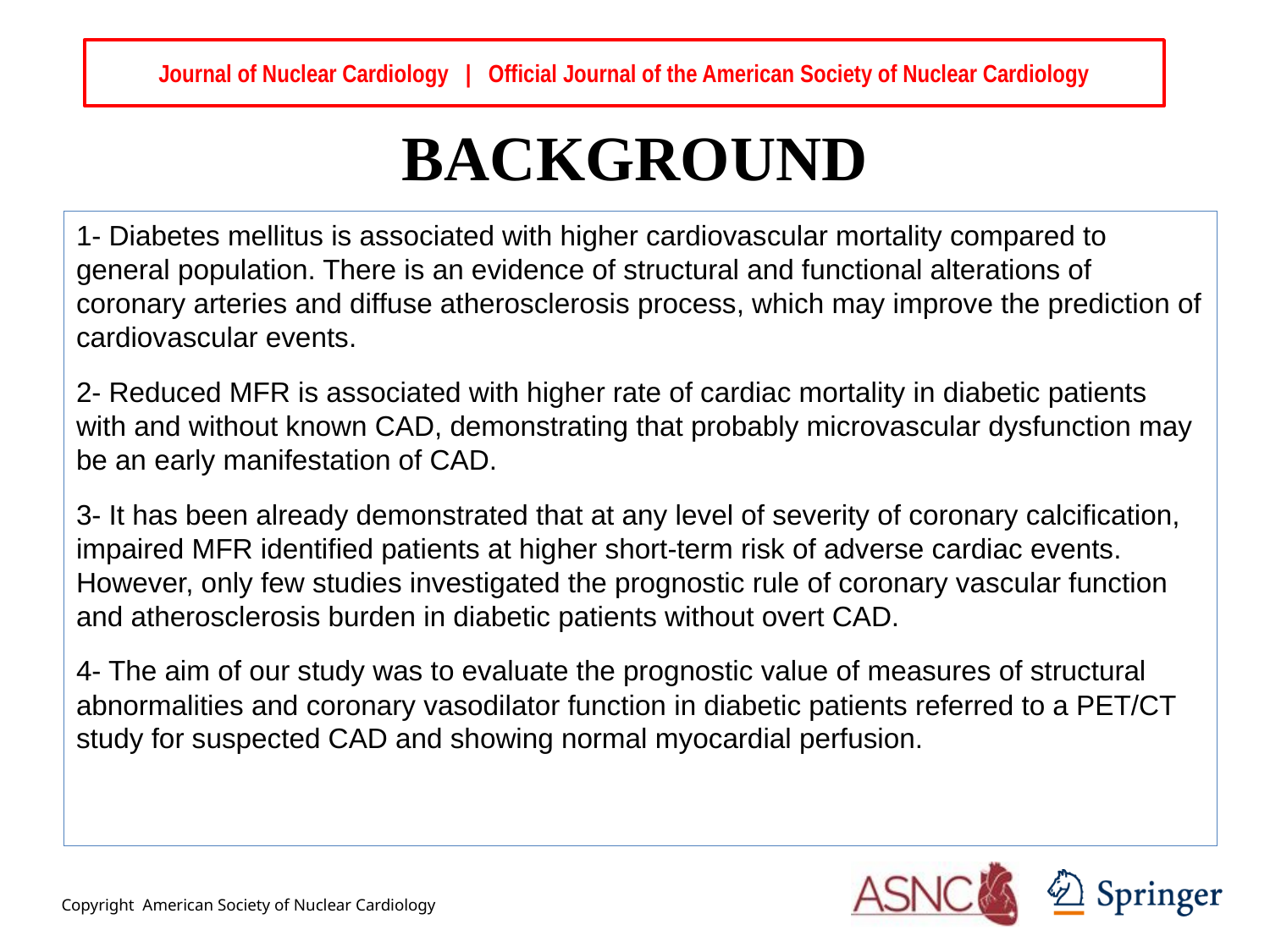

Journal of Nuclear Cardiology | Official Journal of the American Society of Nuclear Cardiology
# BACKGROUND
1- Diabetes mellitus is associated with higher cardiovascular mortality compared to general population. There is an evidence of structural and functional alterations of coronary arteries and diffuse atherosclerosis process, which may improve the prediction of cardiovascular events.
2- Reduced MFR is associated with higher rate of cardiac mortality in diabetic patients with and without known CAD, demonstrating that probably microvascular dysfunction may be an early manifestation of CAD.
3- It has been already demonstrated that at any level of severity of coronary calcification, impaired MFR identified patients at higher short-term risk of adverse cardiac events. However, only few studies investigated the prognostic rule of coronary vascular function and atherosclerosis burden in diabetic patients without overt CAD.
4- The aim of our study was to evaluate the prognostic value of measures of structural abnormalities and coronary vasodilator function in diabetic patients referred to a PET/CT study for suspected CAD and showing normal myocardial perfusion.
Copyright American Society of Nuclear Cardiology

## Slide 3
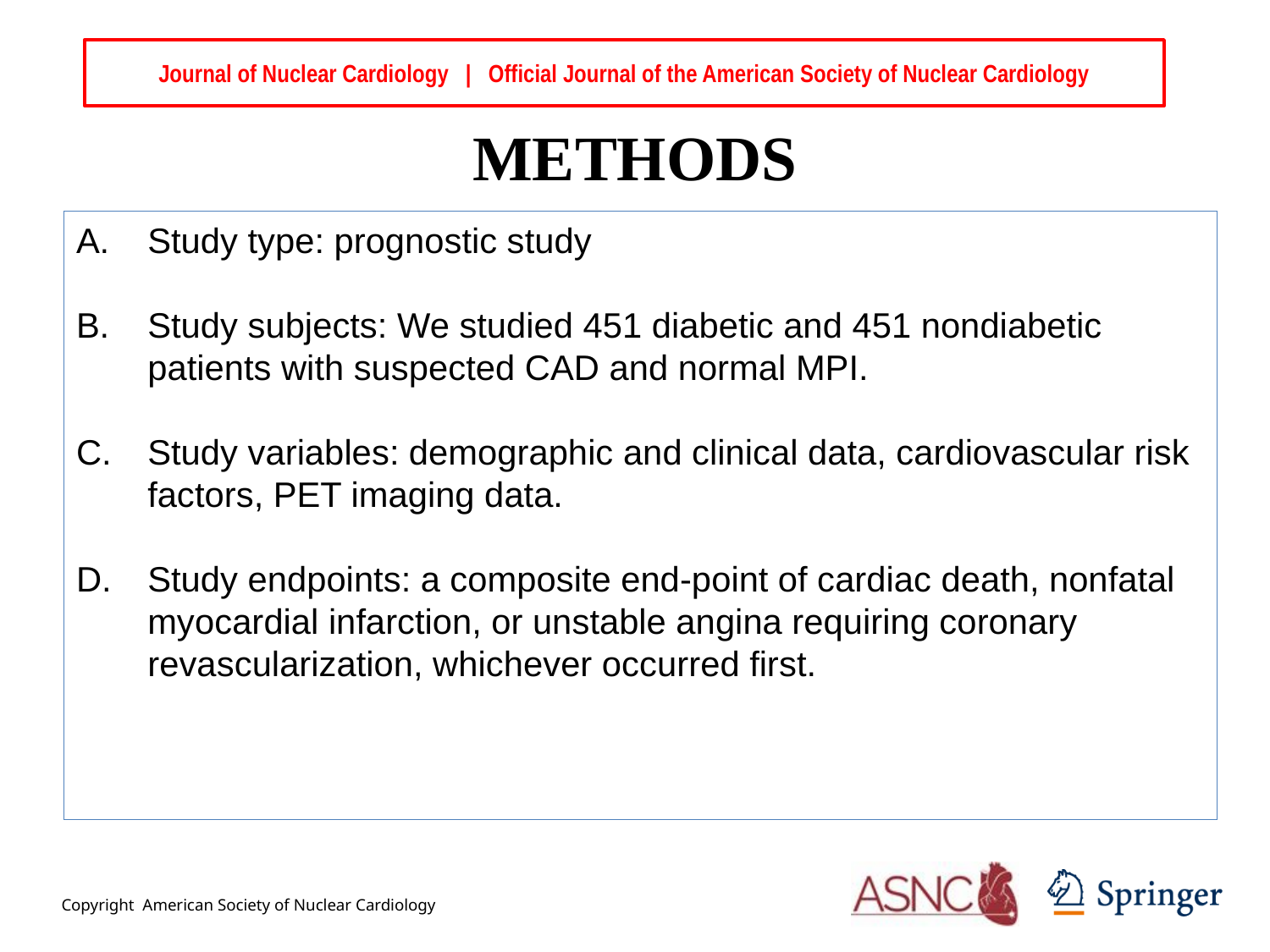

Journal of Nuclear Cardiology | Official Journal of the American Society of Nuclear Cardiology
# METHODS
Study type: prognostic study
Study subjects: We studied 451 diabetic and 451 nondiabetic patients with suspected CAD and normal MPI.
Study variables: demographic and clinical data, cardiovascular risk factors, PET imaging data.
Study endpoints: a composite end-point of cardiac death, nonfatal myocardial infarction, or unstable angina requiring coronary revascularization, whichever occurred first.
Copyright American Society of Nuclear Cardiology

## Slide 4
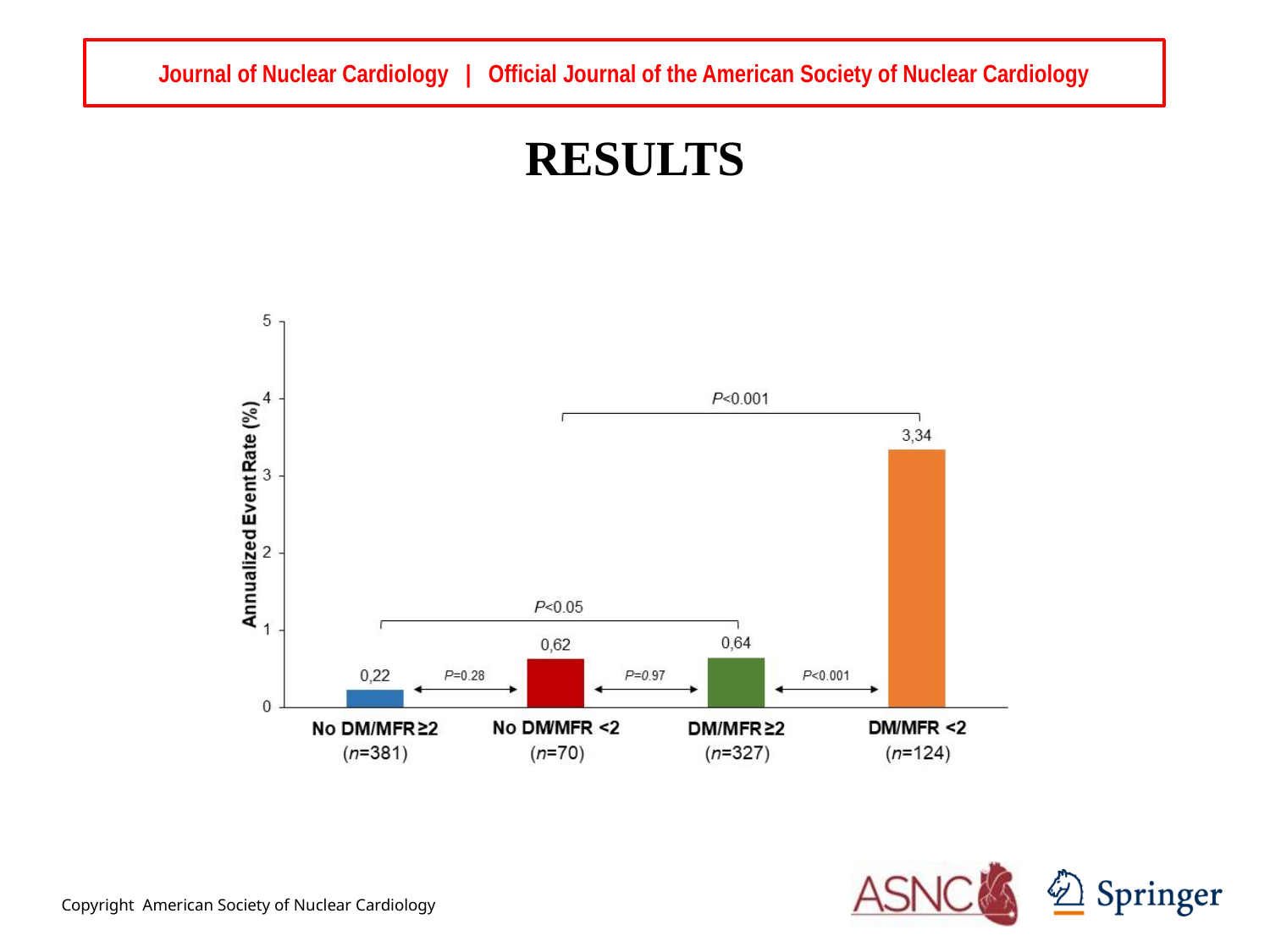

Journal of Nuclear Cardiology | Official Journal of the American Society of Nuclear Cardiology
# RESULTS
Copyright American Society of Nuclear Cardiology

## Slide 5
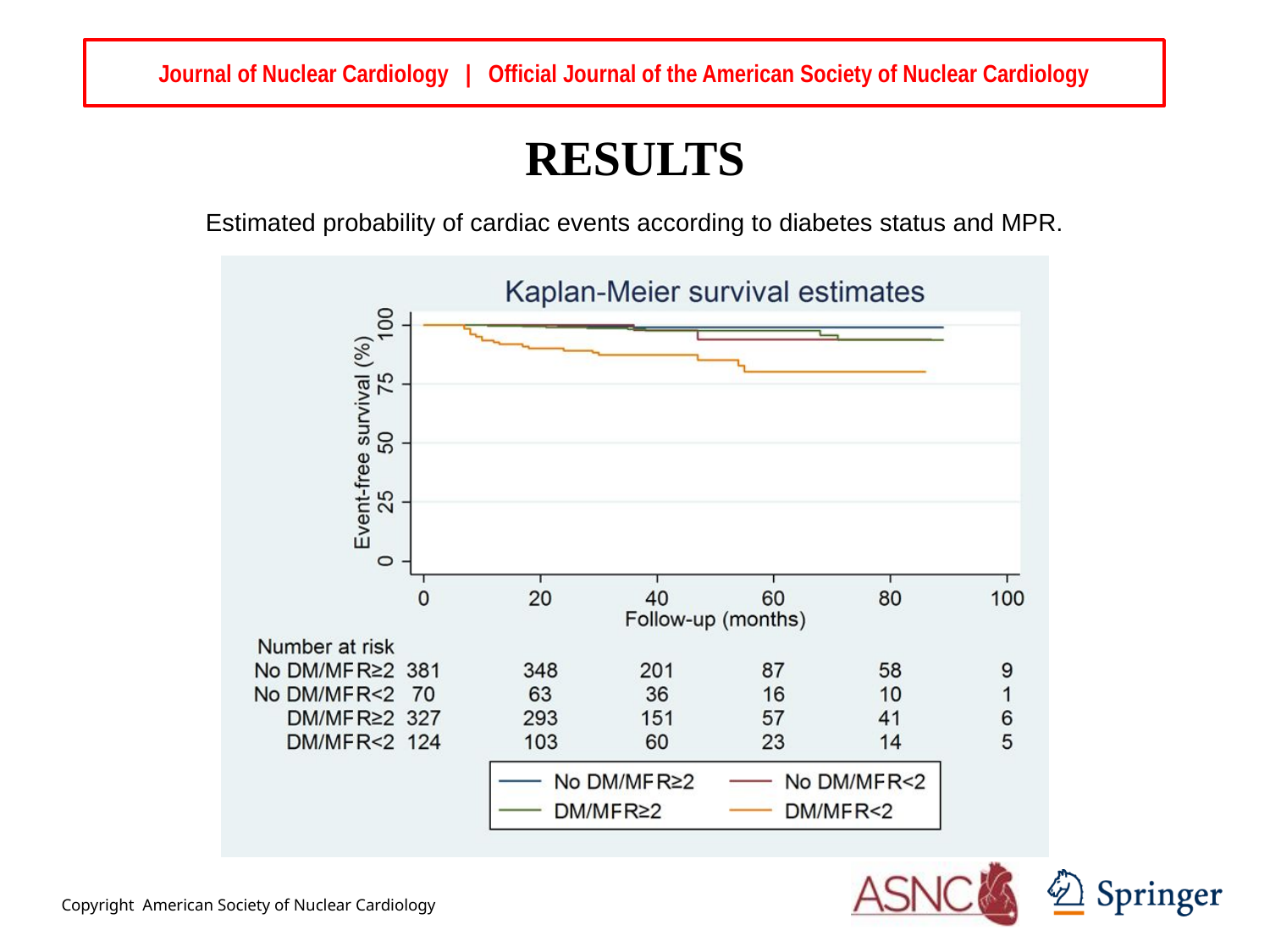

Journal of Nuclear Cardiology | Official Journal of the American Society of Nuclear Cardiology
# RESULTS
Estimated probability of cardiac events according to diabetes status and MPR.
Copyright American Society of Nuclear Cardiology

## Slide 6
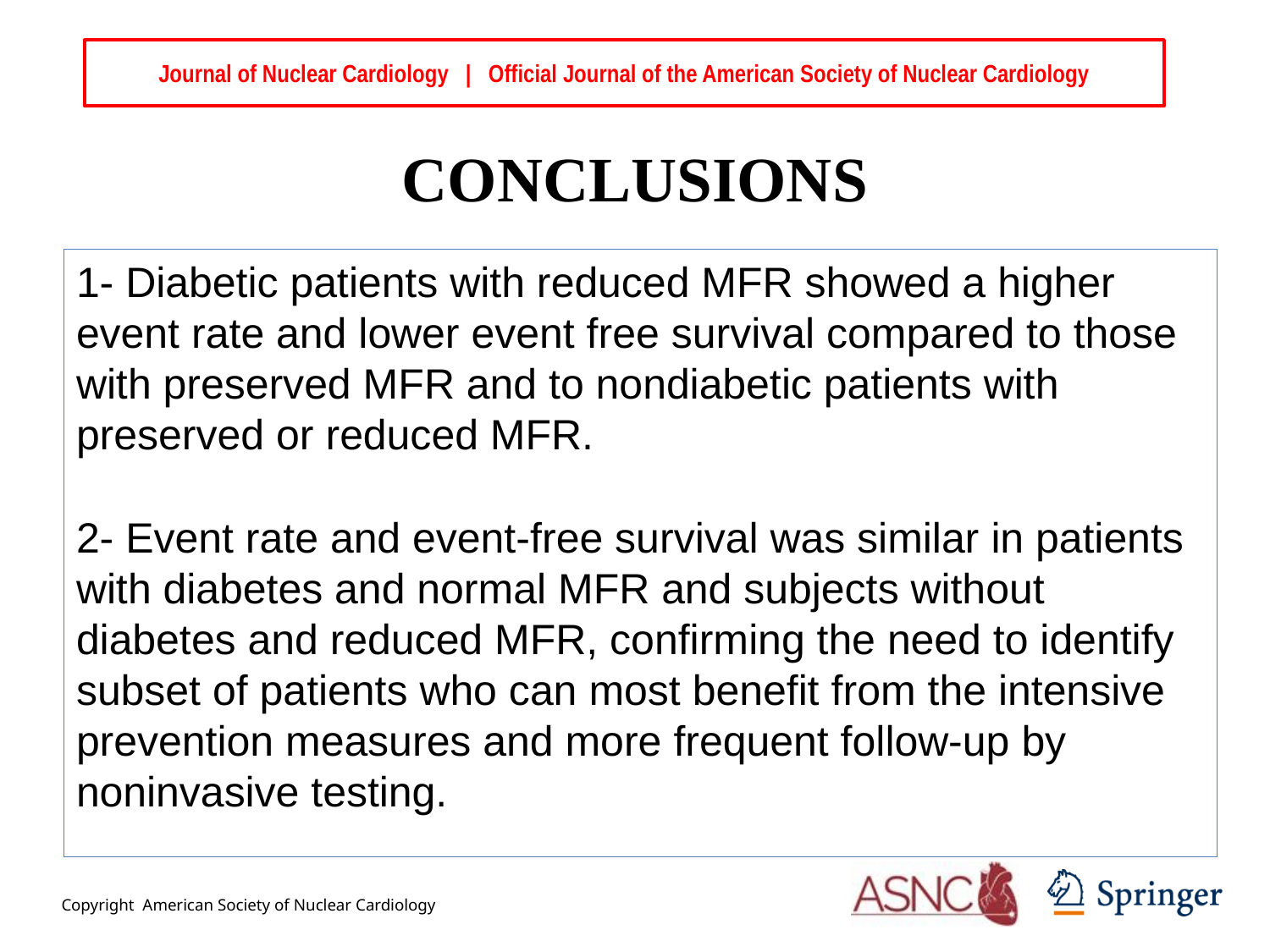

Journal of Nuclear Cardiology | Official Journal of the American Society of Nuclear Cardiology
# CONCLUSIONS
1- Diabetic patients with reduced MFR showed a higher event rate and lower event free survival compared to those with preserved MFR and to nondiabetic patients with preserved or reduced MFR.
2- Event rate and event-free survival was similar in patients with diabetes and normal MFR and subjects without diabetes and reduced MFR, confirming the need to identify subset of patients who can most benefit from the intensive prevention measures and more frequent follow-up by noninvasive testing.
Copyright American Society of Nuclear Cardiology
